# Developmental and Molecular Insights into AgNO_3_-Induced Partial Masculinization and Stamen Abortion in *Siraitia grosvenorii* C. Jeffrey ex A.M. Lu and Zhi Y. Zhang

**DOI:** 10.3390/plants15182796

**Published:** 2026-09-11

**Authors:** Yan Wang, Yanyan Pei, Keke Ning, Jiahe Ning, Gang Li, Shixiang Wang, Qiong Zhou, Yunchuan Mo

**Affiliations:** 1 College of Agriculture, Guangxi University, Nanning 530004, China; 2 Maize Research Institute of Guangxi Academy of Agricultural Science, Nanning 530007, China; 3 Plant Protection and Quarantine Station of Bobai County, Yulin 537600, China; 4 Guangxi Medicinal Botanical Garden, Nanning 530023, China; 5 Agricultural Service Center of Yueli Town, Nandan County, Hechi 547200, China

**Keywords:** *Siraitia grosvenorii*, silver nitrate (AgNO_3_), partial masculinization, pollen abortion

## Abstract

This study focuses on *Siraitia grosvenorii*, an economically important crop endemic to Guangxi Province, China, with significant value in both the pharmaceutical and food industries. However, its dioecious reproductive system results in low natural pollination efficiency, necessitating reliance on costly artificial pollination in commercial production, which severely constrains large-scale cultivation and the breeding of improved varieties. Herein, we systematically investigated the molecular mechanisms of silver nitrate (AgNO_3_)-induced partial masculinization of female flowers, resulting in the formation of morphologically bisexual flowers with aborted stamens. Morphological observations revealed that untreated female flowers exhibited permanently arrested stamen primordia, whereas spraying treatments with 0–300 mg/L AgNO_3_ triggered the development of morphologically complete bisexual flowers. Nevertheless, AgNO_3_-induced stamens exhibited severely disrupted male reproductive development, as indicated by the absence of mature pollen grains at anthesis. Cytological evidence demonstrated that pollen abortion was initiated at the pollen mother cell (PMC) stage, accompanied by abnormal vacuolation and impaired nutrient metabolism in tapetal cells. The dominant abortive phenotype was that PMCs exhibited abnormal development and progressive degeneration during the meiotic developmental window. Methylation-sensitive amplification polymorphism (MSAP) assays further revealed markedly elevated CpG-type methylation levels in AgNO_3_-induced bisexual flowers at early developmental stages. Expression analysis revealed that *SgWIP1* exhibited distinct temporal expression patterns among female, male, and AgNO_3_-induced bisexual flowers, with reduced expression during later stages of stamen development in induced bisexual flowers. Collectively, our findings confirm that AgNO_3_ only partially activates the stamen developmental cascade in *S. grosvenorii.* Extensive epigenetic reprogramming triggered by AgNO_3_, together with the inability to sustain the expression of core male-determining genes, is tightly linked to tapetal dysfunction and subsequent pollen sterility. This study aims to elucidate the unique mechanism by which AgNO_3_-induced partial masculinization in *S. grosvenorii* from multiple perspectives—including tissue and cellular structure, DNA methylation epigenetic modifications, and gene transcription regulation—using techniques such as morphoanatomy, histochemistry, epigenetics (MSAP), and gene expression analysis. The findings of this study will contribute to molecular breeding efforts focused on sex regulation and promote industrial development.

## 1. Introduction

*Siraitia grosvenorii* is a unique medicinal and edible plant endemic to Guangxi, China. Its fruits are abundant in bioactive compounds such as mogrosides, which confer potent anti-allergic, anti-cancer and antibacterial bioactivities [1]. Fruit extracts also serve as efficient scavengers of reactive oxygen species in vitro and can alleviate the decline in antioxidant enzyme activities in alloxan-induced mouse models of type 1 diabetes [2,3], giving this crop immense economic and medicinal value. However, *S. grosvenorii* is a strictly dioecious plant, and its large-scale cultivation relies heavily on costly and inefficient artificial pollination, which severely impedes its industrial development. Therefore, elucidating its sex determination mechanisms, exploring strategies for modulating sex differentiation, and developing bisexual germplasm are of critical scientific importance and practical value for overcoming the pollination bottleneck and enabling varietal improvement.

Plant sexual differentiation is governed by an intricate regulatory network integrating genetic determinants, phytohormones and environmental signals [4]. Sex determination is not a rigid genetic program but exhibits remarkable developmental plasticity. Ethylene, a pivotal plant hormone, acts as a central regulatory hub modulating sexual plasticity via its biosynthetic cascade [5]. Two rate-limiting enzymes sequentially catalyze ethylene biosynthesis: 1-aminocyclopropane-1-carboxylate synthase (ACS) converts S-adenosylmethionine (SAM) to the intermediate ACC, followed by 1-aminocyclopropane-1-carboxylate oxidase (ACO) that oxidizes ACC into ethylene [6].

Precise modulation of ethylene biosynthesis is indispensable for sex determination across cucurbit species, and ethylene itself rather than its precursor ACC dominates the regulatory function. For instance, loss-of-function mutation of *ACO2* disrupts carpel development and triggers a transition from female to male flowers in cucumber [7]. Similarly, downregulated activity of *CpACO1A* transforms female flowers into bisexual flowers in summer squash (*Cucurbita pepo*) [8]. Cumulative evidence indicates that *ACO*-dependent ethylene production serves as a core switch governing floral organ sexual fate, whereas ACS primarily supplies ACC substrates for this pathway. In cucurbits, ethylene signaling directly shapes floral sex identity through a regulatory cascade centered on the ACS gene family and WIP1-type transcription factors.

Silver nitrate (AgNO_3_) is a widely used chemical regulator of floral sex expression because of its ability to interfere with ethylene perception. In cucurbit species, ethylene acts as a central hormonal signal promoting female flower development, whereas suppression of ethylene signaling can release the inhibitory effect on stamen development and shift floral identity toward male or bisexual phenotypes [9,10,11]. However, whether similar regulatory processes occur in dioecious *S. grosvenorii* remains unclear. Previous studies have demonstrated that AgNO_3_ treatment can effectively induce male flowers or morphological bisexual flowers in several monoecious cucurbit crops, including cucumber and other Cucurbitaceae species [12]. These findings suggest that AgNO_3_ provides a valuable physiological approach for dissecting the regulatory plasticity of floral sex determination. However, whether AgNO_3_-mediated ethylene inhibition can similarly regulate sexual differentiation in dioecious cucurbit species remains unclear. Therefore, in this study, AgNO_3_ was selected as a tool to investigate the regulatory response of female flowers in *S. grosvenorii* and to explore the mechanisms underlying AgNO_3_-induced bisexual flower formation and subsequent stamen developmental arrest. Nevertheless, a critical scientific gap remains unresolved: whether the regulatory cascade of AgNO_3_-induced partial masculinization differs between dioecious and monoecious cucurbit species. Our preliminary phenotypic observations demonstrated that AgNO_3_ could induce bisexual flowers from female buds of *S. grosvenorii*, yet the induced stamens were fully abortive, implying a species-specific regulatory bottleneck unique to this dioecious crop. To date, systematic investigations focusing on how AgNO_3_ treatment reshapes cytological progression, epigenetic landscape and transcriptional networks of core sex-determining genes in *S. grosvenorii* floral organs are still absent. Therefore, this study aimed to investigate the developmental and molecular basis of AgNO_3_-induced bisexual flower formation in dioecious *S. grosvenorii*. First, morphological analyses were performed to determine whether AgNO_3_ could induce stamen initiation in female flowers and characterize the resulting floral phenotypes. Histological observations were subsequently conducted to identify the developmental stage and cellular characteristics associated with stamen abortion. Artificial pollination and fruit set analyses were performed to determine whether female reproductive capacity was retained in AgNO_3_-induced bisexual flowers. Furthermore, qRT-PCR was used to examine the expression patterns of candidate sex-related regulatory genes, including *SgWIP1* and *SgACS* family members, while MSAP analysis was conducted to investigate CpG-type methylation alterations associated with AgNO_3_-induced floral developmental changes. Together, these approaches provide insights into how AgNO_3_ uncouples stamen initiation from functional male fertility in dioecious *S. grosvenorii*. This study aims to elucidate the unique mechanism by which AgNO_3_-induced partial masculinization in *S. grosvenorii* from multiple perspectives—including tissue and cellular structure, DNA methylation epigenetic modifications, and gene transcription regulation—using techniques such as morphoanatomy, histochemistry, epigenetics (MSAP), and gene expression analysis. The findings of this study will contribute to molecular breeding efforts focused on sex regulation and promote industrial development.

## 2. Results

### 2.1. AgNO_3_ Induced Continued-Development Stamen in Female Flowers

**Figure 1:**
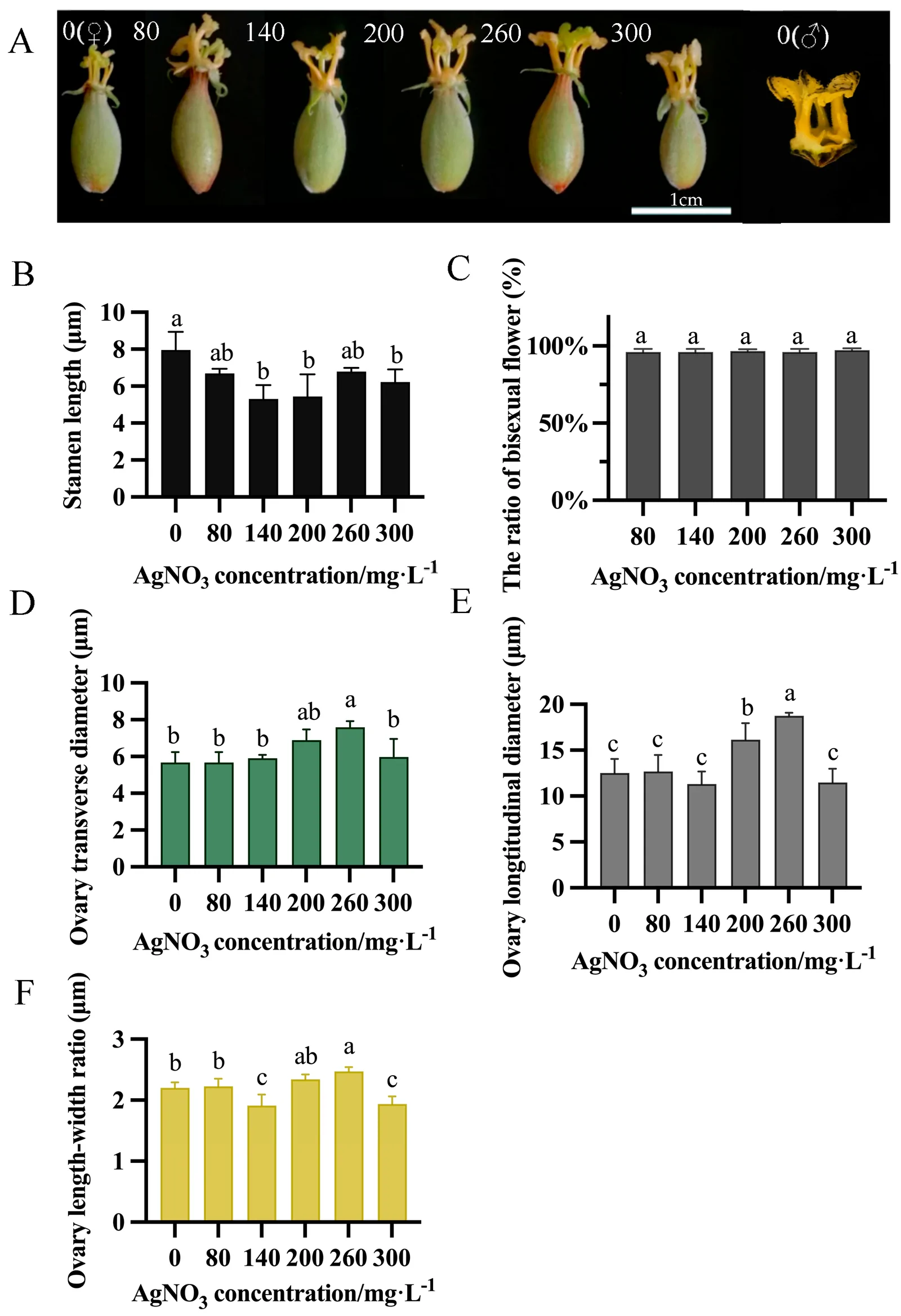
Floral phenotypes (**A**), stamen length (**B**), success rate of bisexual flower induction (**C**), transverse diameter of the ovary (**D**), longitudinal diameter of the ovary (**E**), and the ratio of the longitudinal to transverse diameter of the ovary (**F**) during the flowering stage, 15 days after treatment with different concentrations of AgNO_3_. In (**A**), 0 (♀) represents female flowers treated with 0 mg/L AgNO_3_; 80–300 represents bisexual flowers induced by 80–300 mg/L AgNO_3_; 0 (♂) represents male flowers treated with 0 mg/L AgNO_3_. The data in Subfigure (**C**) were obtained from three independently selected plants, with 50 flowers randomly selected from each plant to count the number of bisexual flowers and calculate the proportion of bisexual flowers; The data in Subfigures (**D**–**F**) were obtained from three randomly selected independent plants, with two flowers randomly selected from each plant. Error bars represent the standard deviation (SD). Statistical significance was assessed using one-way analysis of variance (ANOVA) followed by Tukey’s HSD test. Different lowercase letters above the error bars indicate significant differences at the *p* < 0.05 level.

Under 0 mg/L AgNO_3_, stamen development ceased at the filament elongation stage. In contrast, treatment with 80–300 mg/L AgNO_3_ induced morphologically normal stamens in female flowers, with bisexual flower induction rates of 96.0%, 96.0%, 96.7%, 96.0%, and 97.3% for the 80, 140, 200, 260, and 300 mg/L concentrations, respectively (Figure 1A–C). Although the stamens formed via AgNO_3_ induction were shorter than those of untreated wild-type male flowers, AgNO_3_ application exerted no inhibitory effects on ovary growth and did not trigger any visible morphological defects in ovaries (Figure 1A,B). Treatments with 200 mg/L and 260 mg/L AgNO_3_ significantly elongated ovaries, and the 260 mg/L treatment further markedly enlarged ovary width (Figure 1D,E). Moreover, ovaries from the 200 mg/L and 260 mg/L AgNO_3_ groups possessed a larger length–width ratio than the control group, with the maximum ratio recorded under 260 mg/L AgNO_3_ treatment (Figure 1F). Collectively, these phenotypic data demonstrate that AgNO_3_ facilitates uninterrupted stamen differentiation in female flowers while maintaining normal ovary development, and even accelerates longitudinal ovary expansion.

### 2.2. AgNO_3_ Treatment Exerts No Adverse Effects on Megasporogenesis and Ovary Fruit Set

**Figure 2:**
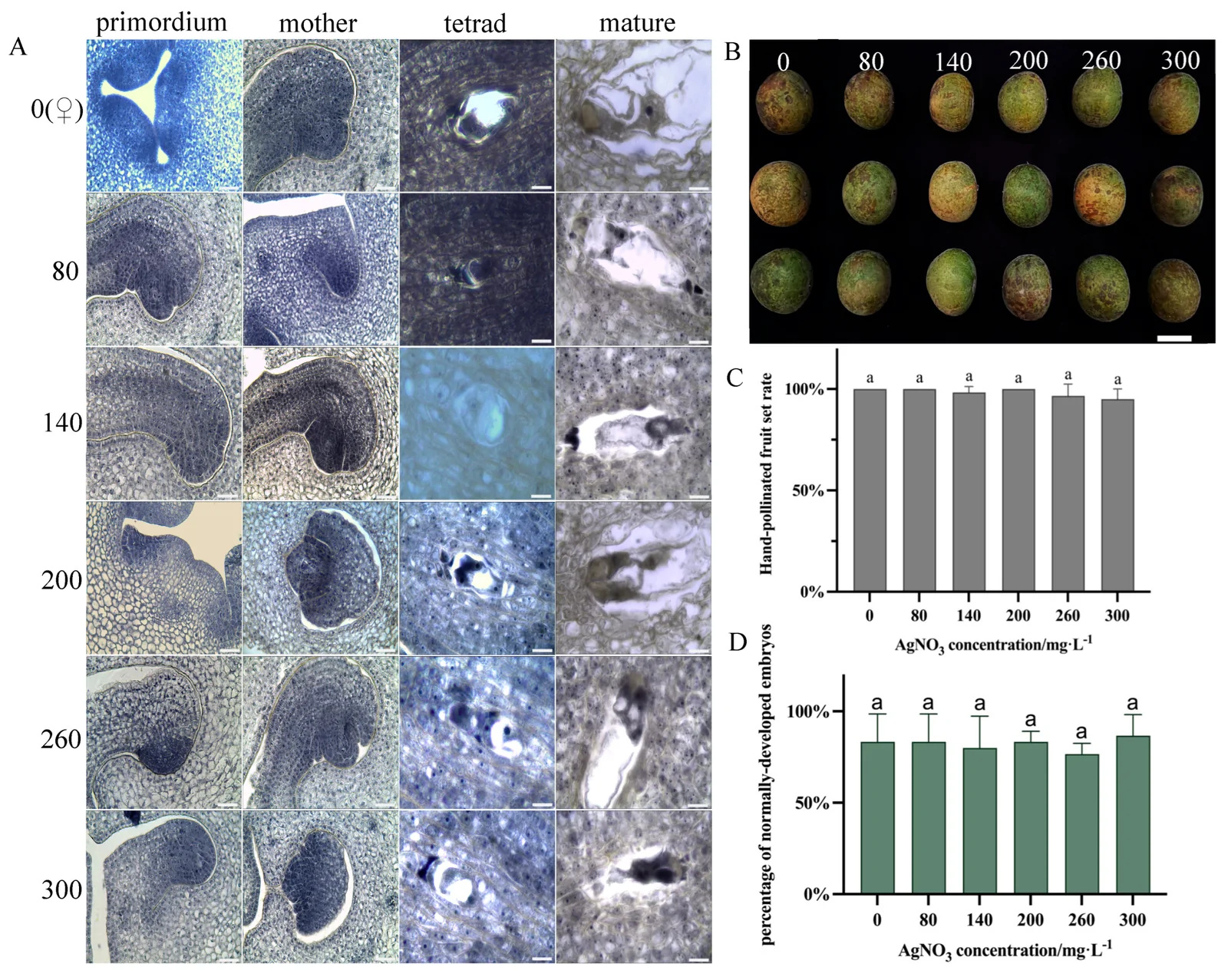
Development of megaspores in female and bisexual flowers treated with AgNO_3_ (**A**); fruit phenotype 30 days after pollination (**B**); fruit set rate (**C**); the proportion of seeds with normal embryos per fruit (**D**). In Subfigure (**A**), 0 (♀) indicates female flowers treated with 0 mg/L AgNO_3_; 80–300 denotes bisexual flowers induced by 80–300 mg/L AgNO_3_; each image represents 9 cross-sections from 3 flowers, with a scale bar of 40 μm. In Subfigure (**B**), each treatment shows three fruits from three independent plants, with a scale bar of 5 cm. The values in Subfigure (**C**) were obtained from 23 flowers selected from three independent plants. Subfigure (**D**) displays 10 embryos selected from each fruit; three fruits were selected from each treatment for statistical analysis. All samples in the figure were randomly selected. Error bars represent the standard deviation (SD). Statistical significance was assessed using one-way analysis of variance (ANOVA) followed by Tukey’s HSD test. Different lowercase letters above the error bars indicate significant differences at the *p* < 0.05 level.

As illustrated in Figure 2, untreated female flowers (0 mg/L AgNO_3_) formed Polygonum-type embryo sacs. The megaspore mother cell underwent meiotic division to generate a megaspore tetrad, followed by successive mitoses that yielded a mature seven-celled, eight-nucleate embryo sac containing three antipodal cells, two synergids, one egg cell, and a central cell (Figure 2A). Flowers exposed to 80–300 mg/L AgNO_3_ exhibited identical embryo sac developmental patterns to that of the control group. Artificial pollination with pollen collected from untreated male flowers revealed that all AgNO_3_-treated female flowers could successfully set fruit, with no statistically significant intergroup disparities in fruit dimensions and the stage of embryo development in the seeds of the fruit (Figure 2B–D). Combining embryo sac developmental observations with ovary transverse diameter gradients, we categorized ovary development into four distinct stages. (1) Ovary transverse diameter < 3.0 mm: embryo sac primordium stage; (2) 3.0–5.0 mm: megaspore mother cell and tetrad stages; (3) 5.0–7.0 mm: binucleate-to-tetranucleate embryo sac stage; (4) >7.0 mm: mature embryo sac stage (Table S1).

### 2.3. AgNO_3_ Triggers Pollen Mother Cell (PMC) Abortion in Induced Stamens

**Figure 3:**
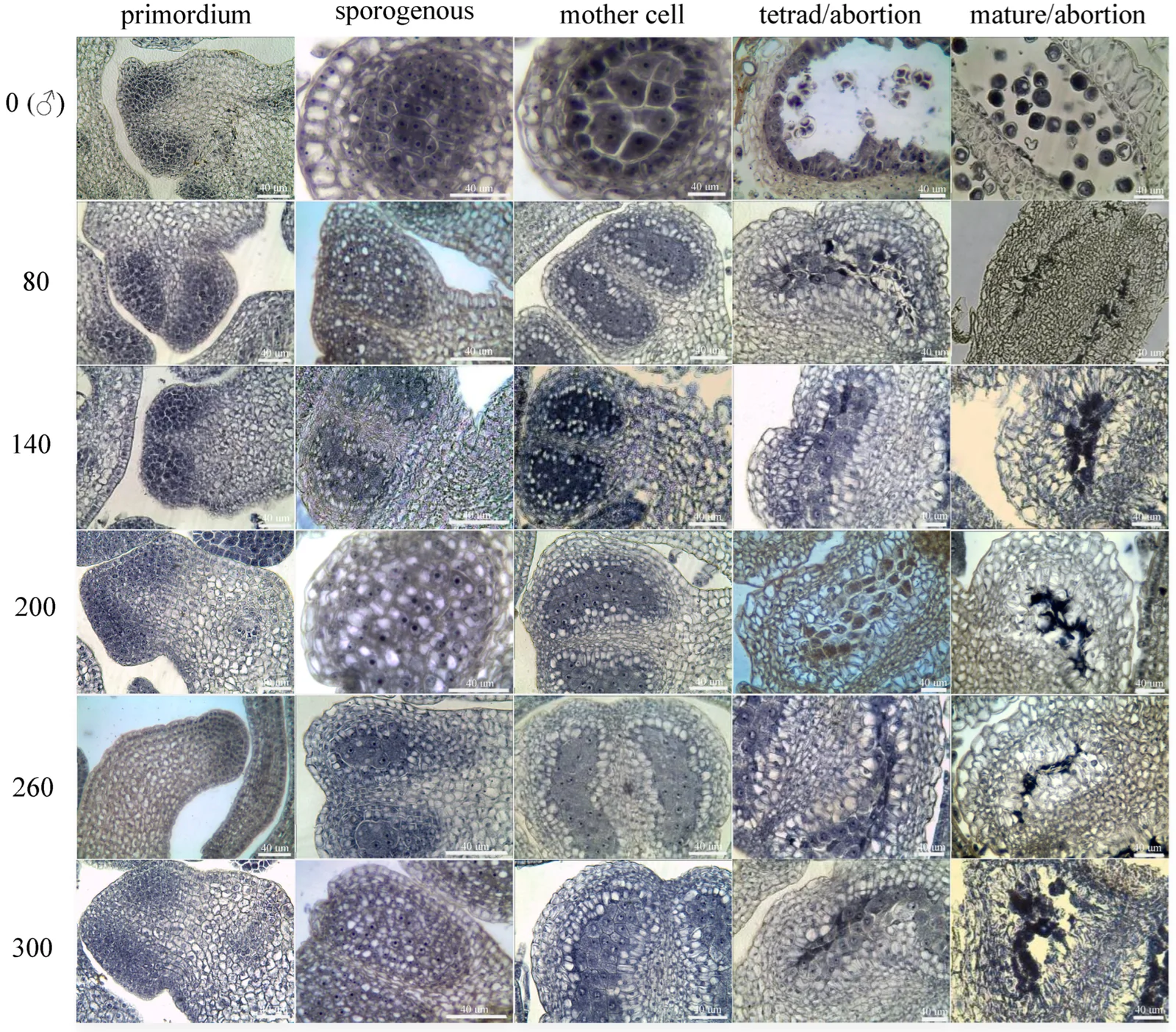
Development of microspores in male flowers and AgNO_3_-induced bisexual flowers. “0 (♂)” refers to anthers collected from male plants treated with 0 mg/L AgNO_3_; “80–300” refers to anthers from AgNO_3_-induced bisexual flowers treated with 80–300 mg/L AgNO_3_, respectively. Each image represents nine sections from nine flower buds collected from three randomly selected independent plants, with a scale bar of 40 μm.

As displayed in Figure 3, the central cells within anther primordia of untreated male flowers (0 mg/L AgNO_3_) expanded with condensed nucleocytoplasm and progressively differentiated into sporogenous cells and pollen mother cells (PMCs). Concurrent with this differentiation, the monolayer tapetum expanded and accumulated abundant nutritive substances. Subsequent meiosis of PMCs generated microspore tetrads; these microspores then developed from uninucleate pollen into mature trinucleate pollen grains, while the tapetum gradually degenerated and vanished. In stark contrast, although enlarged PMC-like cells with condensed nucleoplasm emerged in the central region of anther primordia at early floral developmental stages in stamens induced by 80–300 mg/L AgNO_3_, these cells displayed severe vacuolation, and the surrounding tapetum differentiated into 1–3 layers of heavily vacuolated cells. As floral development proceeded, PMCs in AgNO_3_-induced stamens degenerated progressively, failed to complete regular meiosis, and ultimately produced the absence of mature pollen grains at anthesis. Intriguingly, partial PMCs in stamens treated with 200 mg/L AgNO_3_ accomplished one round of meiosis to form dyad-like cellular structures; nevertheless, such dyads could not sustain normal development and finally degraded entirely.

Combining cytological observations of microspore development and measurements of bud transverse diameter, we classified anther development of untreated male flowers (0 mg/L AgNO_3_) into five distinct phases: bud diameter < 1.00 mm for the anther primordium and sporogenous cell stage, 1.00–1.50 mm for the PMC stage, 1.50–2.50 mm for the tetrad and early uninucleate pollen stage, 2.50–4.50 mm for the late uninucleate pollen stage, and >4.50 mm for the mature anther stage. By analogy, we established a three-stage developmental partition for anthers of AgNO_3_-induced bisexual flowers according to anther developmental traits paired with ovary transverse diameter gradients, including the anther primordium stage at ovary transverse diameter < 3.0 mm, the PMC stage at 3.0–5.0 mm, and a phase featuring progressive degeneration of PMCs when the ovary transverse diameter exceeded 5.0 mm (Table S2).

### 2.4. AgNO_3_ Triggers CpG-Type Methylation DNA Hypermethylation in Female Flowers of S. grosvenorii

MSAP profiling yielded distinct, repeatable band patterns for every primer pair (Figure S1). In total, 50 primer combinations amplified 4398 readable fragments, among which 2171 were derived from female flowers and 2227 from AgNO_3_-induced bisexual flowers.

For female flowers, we detected 348 (16.03%) loci with internal cytosine methylation, 231 (10.64%) loci with hemimethylation or internal cytosine methylation, 29 (1.34%) loci showing complete double-stranded methylation, and 1563 (71.99%) unmethylated loci. By contrast, bisexual flowers contained 380 (17.06%) loci with internal cytosine methylation, 182 (8.17%) loci with hemimethylation or internal cytosine methylation, 119 (5.34%) loci showing complete double-stranded methylation, and 1546 (69.42%) unmethylated loci (Table 1). The overall methylation ratio detected by MSAP was higher in AgNO_3_-induced bisexual flowers (30.58%) than in female flowers (28.01%). (Table S3).

Comparisons of cytosine methylation profiles at shared loci between female and AgNO_3_-induced bisexual flowers uncovered 20 distinct patterns of differential methylation modification across the two floral phenotypes (Table 1). Frequency statistics of these methylation transitions revealed that bisexual flowers possessed over 500 newly generated CpG-type de novo methylated loci compared with untreated female flowers. Of these differentially modified loci, more than 85 exhibited a shift from the internal CmCGG methylation motif to the mCCGG pattern (Table S4).

### 2.5. AgNO_3_ Reshapes the Differential Expression of Sex-Determining Genes

**Figure 4:**
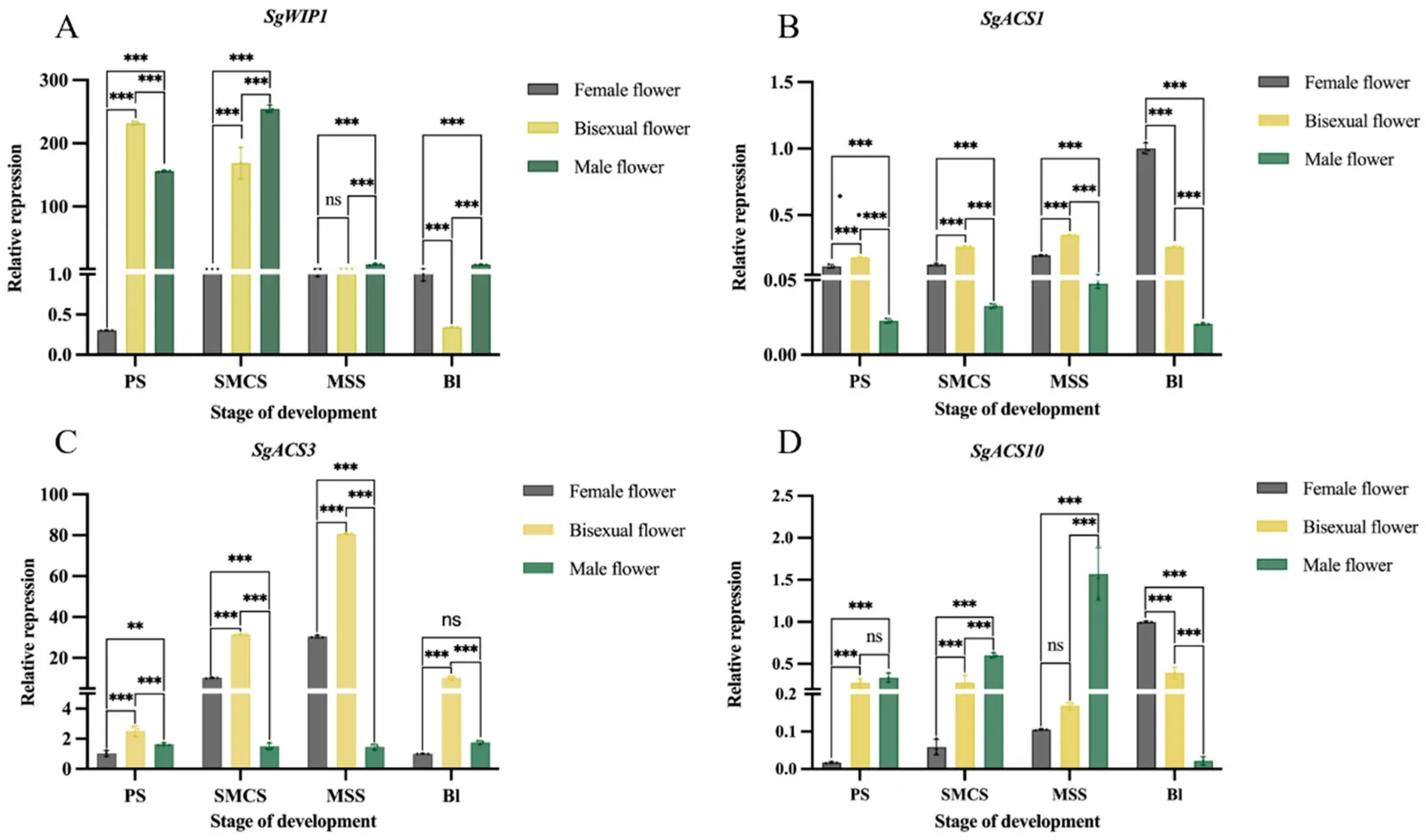
Relative transcript abundances of *SgWIP1*, *SgACS1*, *SgACS3* and *SgACS10* throughout floral development in *Siraitia grosvenorii*. Relative transcript abundance of *SgWIP*1 (**A**); Relative transcript abundance of *SgACS1* (**B**); Relative transcript abundance of *SgACS3* (**C**); Relative transcript abundance of *SgACS10* (**D**). PS, primordium stage; SMCS, spore mother cell stage; MSS, mature spore stage; BI, blooming day. Asterisks denote statistically significant differences (** *p* < 0.01; *** *p* < 0.001).

During floral development, *SgWIP1* displayed stage-dependent expression patterns among female, male, and AgNO_3_-induced bisexual flowers (Figure 4A). Its expression increased during the early stages associated with stamen initiation and was higher in male and induced bisexual flowers than in female flowers. However, *SgWIP1* transcript levels decreased during later developmental stages, including microspore maturation and anthesis, indicating that *SgWIP1* expression is dynamically regulated during stamen development.

The transcript abundance of genes in the *SgACS* family, which are involved in core ethylene biosynthesis, exhibited significant between-group differences across different developmental stages of female, bisexual, and male flowers; specifically, *SgACS1*, *SgACS3*, and *SgACS10* displayed distinct temporal expression patterns. In female flowers, *SgACS1* transcripts accumulated gradually over floral development: its expression rose moderately at early stages and surged sharply on the day of anthesis. By comparison, *SgACS1* expression stayed relatively constant throughout embryo sac formation in bisexual flowers, with lower transcript quantities than those in female flowers at early developmental phases. Conversely, *SgACS1* maintained weak transcription during microspore development in male flowers (Figure 4B). Collectively, these transcriptomic data indicate that *SgACS1* expression is positively correlated with late-stage embryo sac maturation in female *S. grosvenorii*. The higher *SgACS1* abundance in bisexual flowers may participate in modulating male floral organ differentiation, while exogenous AgNO_3_ application tends to drive the upregulation of this gene during floral morphogenesis.

During spore developmental progression, transcript abundances of *SgACS3* gradually climbed in both female and AgNO_3_-induced bisexual flowers before dropping drastically at anthesis, and bisexual flowers retained markedly higher *SgACS3* transcript levels than female flowers at all tested stages. On the contrary, male flowers exhibited constitutively weak *SgACS3* transcription throughout the entire microsporogenesis process (Figure 4C). These transcriptional observations demonstrate that *SgACS3* expression is positively correlated with embryo sac maturation in *S. grosvenorii*, and this gene may cooperate with other regulatory factors to coordinate male floral organ development.

At all stages of spore development, *SgACS10* maintained high transcript abundance in both AgNO_3_-induced bisexual and male flowers; in contrast, *SgACS10* expression levels in female flowers were lower than those in bisexual flowers (Figure 4D). These transcriptional profiles reveal that robust *SgACS10* expression is positively correlated with anther morphogenesis in *S. grosvenorii*, and this gene may collaborate with other genetic regulators to modulate anther development.

## 3. Discussion

### 3.1. AgNO_3_ Breaks the Selective Developmental Arrest of Stamens in Female Flowers and Promotes Stamen Development

This study confirms that AgNO_3_ can induce the development of female flowers into bisexual flowers in dioecious *S. grosvenorii*, but the induced bisexual flowers exhibit male sterility. A similar response has been reported in the dioecious cucurbit *Coccinia grandis*, in which AgNO_3_ treatment promoted stamen development in female flowers but failed to restore pollen fertility [10]. This phenomenon of “morphological formation but functional deficiency”—a “partial reversal”—stands in stark contrast to the results showing that AgNO_3_ can induce fertile bisexual flowers in monoecious plants such as cucumber and melon [13,14], indicating the uniqueness of the sex regulation network in dioecious *S. grosvenorii*. Importantly, the induction of stamen development was not accompanied by significant disruption of female reproductive development.

Examination of paraffin sections revealed that, although AgNO_3_ treatment induced partial masculinization, the embryos in the bisexual flowers induced at all concentrations (80–300 mg/L) completed the normal developmental process (from ovule primordia formation to mature embryo sacs), showing no significant differences compared to untreated female flowers (Figure 2A). This result confirms that AgNO_3_ did not interfere with the normal development of female gametes during the induction of stamen morphogenesis, and that bisexual flowers fully retained female fertility. This further supports the view that the two reproductive developmental programs are not fully coupled in *S. grosvenorii* treated with AgNO_3_.

The cytological abnormalities observed in this study in AgNO_3_-induced bisexual flowers of *S. grosvenorii* provide a plausible cytological explanation for the failure of male functional development. In particular, pronounced vacuolization and structural abnormalities of the tapetum were observed around the PMC/meiotic developmental stage, coinciding with degeneration of the developing male reproductive cells. The tapetum is a metabolically active layer that supports developing male meiocytes and microspores by providing nutrients, proteins, lipids, enzymes, and other materials required for pollen development and pollen-wall formation [15]. Therefore, disruption of tapetal development or function can compromise the subsequent development and survival of microspores. The abnormal tapetal morphology observed in the present study is also consistent with cytological features reported in male-sterile plants. In rice, premature programmed cell death (PCD) of tapetal cells has been directly associated with male sterility and pollen collapse, with vacuolation and other degenerative changes occurring during abnormal tapetal degeneration [16]. Conversely, delayed tapetal PCD can also disrupt pollen development. Genetic studies in rice have shown that defects in the timely degradation of the tapetum are accompanied by abnormal pollen-wall formation and microspore abortion [17,18]. In Arabidopsis, defective tapetal development has likewise been associated with tapetal vacuolization, abnormal pollen-wall formation, and severe pollen developmental defects [19]. Taken together, these observations suggest that the pronounced tapetal vacuolization and subsequent degeneration observed in AgNO_3_-induced bisexual flowers may reflect impaired tapetal function and/or disturbed temporal regulation of tapetal PCD. Such defects could reduce the nutritional and developmental support required by PMCs and developing microspores, thereby contributing to the developmental arrest observed around the PMC/meiotic stage and ultimately to male reproductive failure. However, since this study did not directly evaluate PCD itself, it remains to be further investigated whether AgNO_3_-induced male sterility in bisexual flowers results from premature or delayed initiation of PCD, or from other regulatory abnormalities.

### 3.2. Molecular Association Between Dysregulated Core Gene Expression and Stamen Abortion

In *S. grosvenorii*, the ability of AgNO_3_ to induce stamen formation in female flowers may be related to its effect on ethylene synthesis. Ethylene is the primary hormonal regulator of sex expression in Cucurbitaceae plants—in cucumbers, increased ethylene production promotes the development of female flowers and inhibits stamen development [20]. Consistent with this model, inhibiting ethylene biosynthesis or its action can shift floral organ development toward a male or bisexual phenotype [21]. It has been reported that AgNO_3_ can also induce stamen development in dioecious plants of the Cucurbitaceae family, which suggests that AgNO_3_ may possess ethylene-inhibiting activity [22,23]. This study investigated the expression patterns of *SgACSs*, key genes involved in ethylene biosynthesis in *S. grosvenorii*, following treatment with AgNO_3_. ACS is a core gene family involved in ethylene biosynthesis, and multiple ACS genes have been shown to be directly involved in the regulation of sex expression in Cucurbitaceae plants [24,25,26]. For example, several ACS family members in the Cucurbitaceae family are involved in the regulation of ethylene synthesis: the expression of *CsACS1G*, *CsACS2*, and *CsACS11* in cucumbers increases ethylene production and accumulation [7,27,28,29,30]. Bitter melon (*McACS4*) [31], zucchini (*CpACS27A*) [32], watermelon (*CitACS3*) [31], and pumpkin (*CpACS27A*) [32] also exert sex-specific regulation by controlling ethylene synthesis. The results of this study indicate that there are significant differences in the expression levels of *SgACS1*, *SgACS3*, and *SgACS10* across different floral organs and stages of floral development: *SgACS1* is preferentially expressed in female flowers; during spore development, the transcriptional level of *SgACS3* in bisexual flowers remains consistently higher than that in female flowers, whereas during the development of male microspores, *SgACS3* is maintained at a consistently low level. From the early stage of sporogonial formation through the SMCS, the transcriptional levels of *SgACS10* in bisexual and male flowers were consistently higher than those in female flowers; subsequently, during the MSS, *SgACS10* remained significantly enriched in male flowers, whereas its expression level in bisexual flowers showed no significant difference from that in female flowers. These observations suggest that these three genes may be involved in the process of AgNO_3_-nduced anther formation, with *ACS10* likely playing a more critical role.

It is worth noting that the transcription factor WIP1 has been identified as a key regulator of sex determination in Cucurbitaceae plants; it inhibits the carpel identity gene CRC through histone deacetylation, thereby coordinating male flower development [33]. In melon, WIP1 recruits the co-repressor TOPLESS to inhibit carpel development via histone deacetylation, ultimately promoting male flower development [7,33]. In this study, in AgNO_3_-induced bisexual flowers, *SgWIP1* was highly expressed in bisexual and male flowers but at low levels in female flowers during the early stages of sporogony. During the spore maturation stage, WIP1 expression was significantly higher in male flowers than in female flowers, while in bisexual flowers, there was no significant difference compared to female flowers. This suggests that following AgNO_3_ treatment, the *SgWIP1*-mediated regulatory network may not have been fully activated, which may be related to the observed palisade layer dysfunction and pollen sterility. In summary, AgNO_3_ treatment increased the upregulation of key ethylene synthesis genes—*SgACS1*, *SgACS3*, *SgACS10*, and *SgWIP1*—thereby inhibiting ethylene synthesis and promoting stamen development.

### 3.3. AgNO_3_-Associated DNA Methylation Pattern Changes and Their Potential Involvement in Floral Development

The MSAP results further suggest that epigenetic regulation may be involved in the developmental changes associated with AgNO_3_-induced stamen formation and pollen abortion. Altered DNA methylation patterns have been reported in male-sterile plants, and integrated methylome and transcriptome analyses have identified differential methylation associated with changes in gene expression during pollen abortion [34]. DNA methylation is also dynamically regulated during male reproductive development and may transcriptionally regulate genes involved in pollen development [35]. MSAP analysis in this study revealed that the number of methylated CpG sites in the genomes of bisexual flowers was higher than that in female flowers, indicating that AgNO_3_ treatment can affect the methylation levels of genes in female flowers; this may be a key factor in AgNO_3_-induced stamen development.

Despite the initiation of male floral development, the induced stamens failed to complete functional male reproductive development. The altered expression of sex-related genes and accompanying DNA methylation changes may reflect an incomplete or dysregulated execution of the male developmental program. At the cytological level, this developmental disturbance was accompanied by abnormal tapetal development and degeneration of PMCs around the PMC/meiotic developmental stage, followed by failure of normal microspore and pollen development. Consequently, morphologically developed stamens were formed but did not acquire functional male fertility, resulting in the partial masculinization phenotype observed in AgNO_3_-induced bisexual flowers. Importantly, the present data demonstrate the temporal and phenotypic associations among transcriptional changes, DNA methylation alterations, and male reproductive defects, but do not establish a direct causal sequence among these events. In addition, although we successfully induced bisexual flowers using different concentrations of AgNO_3_, we cannot completely rule out the possibility that AgNO_3_ toxicity contributes to the male sterility observed in these flowers.

AgNO_3_ provides a physiological approach to uncouple stamen initiation from functional male fertility, enabling investigation of the developmental constraints underlying sex differentiation in dioecious *S. grosvenorii*. Nevertheless, several limitations should be considered when interpreting the present findings. First, although cytological observations indicated severe disruption of male reproductive development in AgNO_3_-induced stamens, direct functional assays of pollen viability, pollen germination, anther dehiscence, and controlled pollination using pollen from induced stamens were not performed in the present study. Likewise, successful fruit set following pollination with pollen from normal male flowers provides evidence that female reproductive capacity was retained but does not establish complete female fertility. Future studies incorporating comprehensive pollen and seed fertility assessments will be necessary to further characterize the reproductive competence of AgNO_3_-induced bisexual flowers.

Second, the present cytological analysis was based primarily on paraffin-section morphology and did not include chromosome spreads, DAPI staining, or stage-specific meiotic markers. Although PMC degeneration, abnormal cellular structures, and pronounced tapetal vacuolization indicate severe disruption of male reproductive development around the PMC/meiotic developmental stage, the precise stage of meiotic arrest cannot be established from the present evidence. Moreover, the possibility that Ag^+^ toxicity contributes to the observed tapetal and PMC abnormalities cannot be completely excluded because AgNO_3_-treated fertile male flowers, ionic controls, and alternative ethylene inhibitors were not included. Future experiments incorporating these controls together with stage-resolved meiotic cytology and functional fertility assays will help distinguish disruption of the male developmental program from direct Ag^+^ effects on reproductive tissues.

Third, the molecular and epigenetic findings should be interpreted as associations rather than evidence of direct causality. The present study identified altered temporal expression of *SgWIP1* and *SgACS* family members and MSAP-detectable changes in DNA methylation patterns, but it did not establish a direct regulatory relationship between specific methylation events and the expression of male developmental genes. In particular, the MSAP approach surveys a subset of CCGG sites and does not resolve specific differentially methylated regions or their target genes. In addition, the methylation analysis was performed using flowering-stage whole-flower samples and therefore cannot establish methylation dynamics across successive developmental stages or distinguish changes attributable to the developmental stage or tissue composition. Stage-matched, tissue-specific, and higher-resolution methylation profiling, such as whole-genome bisulfite sequencing, integrated with transcriptome analysis, will be required to determine the temporal and cellular specificity of these epigenetic changes.

**Figure 5:**
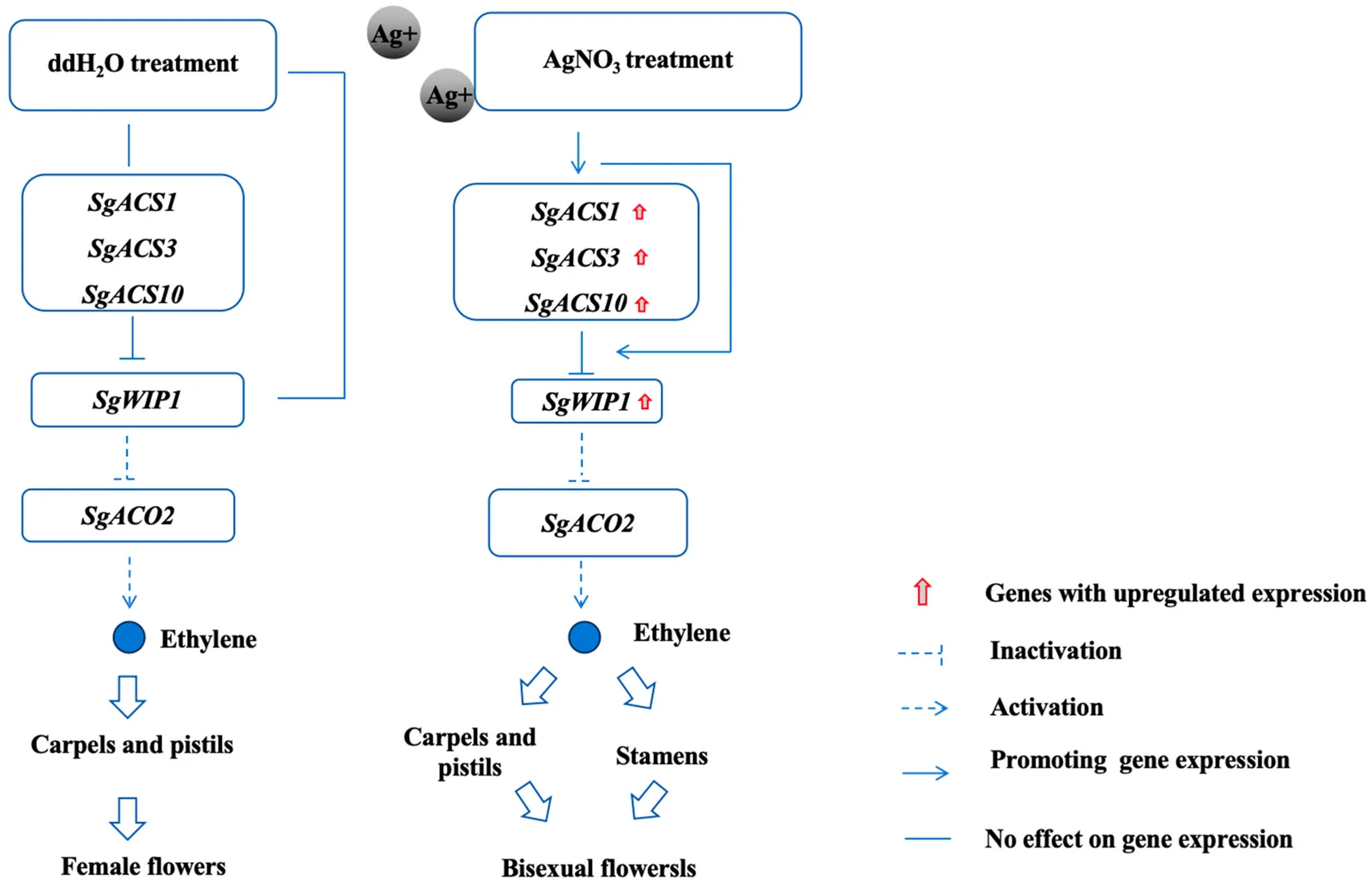
A proposed working model of the mechanism of sex determination in *S. grosvenorii*.

Finally, although the altered expression pattern of *SgWIP1* was associated with incomplete stamen development, its direct functional role in stamen abortion remains to be established. Functional validation through gene overexpression, silencing, or genome editing, together with locus-specific methylation analysis, will be necessary to determine whether changes in *SgWIP1* regulation contribute directly to the observed phenotype. Thus, the present findings support a working model in which AgNO_3_-induced partial masculinization is accompanied by coordinated changes in floral development, sex-related gene expression, and DNA methylation patterns, but the causal relationships among ethylene signaling, epigenetic reprogramming, *SgWIP1* regulation, tapetal dysfunction, and stamen abortion remain to be experimentally determined (Figure 5).

## 4. Materials and Methods

### 4.1. Plant Materials, AgNO_3_ Treatment, and Sampling Methods

This experiment was conducted in Nanning, Guangxi, China, in May. The plant materials consisted of *S. grosvenorii* individuals originally collected from Lingui, Guangxi, China, including untreated female plants, untreated male plants, and female plants subjected to gradient AgNO_3_ treatments. *AgNO_3_* spraying was initiated 50 days after cutting transplantation, when floral bud differentiation was visibly initiated. Female and male plants sprayed with distilled water served as the blank control group. Based on previous studies showing that AgNO_3_ within a range of several tens to several hundred milligrams per liter can effectively interfere with ethylene-mediated floral sex regulation in cucurbit crops, combined with preliminary observations of *S. grosvenorii* floral responses, five AgNO_3_ concentrations (80, 140, 200, 260, and 300 mg/L) were selected for treatment. Female plants sprayed with these AgNO_3_ solutions were designated as the bisexual flower induction groups. Three plants were randomly selected from each treatment group, and their inflorescences were sprayed with AgNO_3_ at 7:00 p.m. each day, with at least 100 flower buds treated per plant. Each inflorescence received 10 mL of solution, and the treated inflorescences were sprayed again 7 days later. No additional shading measures are required after spraying is complete. Floral tissues at successive developmental stages were harvested 15 days after the initial treatment for subsequent cytological and molecular analyses. Female and induced bisexual flowers were divided into six developmental stages based on ovary transverse diameter: 0–1.0 mm, 1.0–2.0 mm, 2.0–3.0 mm, 3.0–4.0 mm, 4.0–5.0 mm, 5.0–6.0 mm, 6.0–7.0 mm, and >7.0 mm. Male flowers were also classified into six developmental stages according to flower bud transverse diameter: 0–1.0 mm, 1.0–1.5 mm, 1.5–2.0 mm, 2.0–2.5 mm, 2.5–3.0 mm, 3.0 mm–4.5 mm and >4.5 mm. Floral developmental stages were determined based on a combination of morphological measurements and cytological characteristics. For female and AgNO_3_-induced bisexual flowers, developmental stages were classified according to ovary transverse diameter and corresponding embryo sac development. For male flowers, developmental stages were determined according to bud diameter and corresponding microspore development. The detailed criteria for each developmental stage are provided in the Supplementary Tables S1–S6.

Experimental design and sampling strategy. Three independent plants were randomly selected for each treatment group and were considered biological replicates. For DNA methylation analysis, one flower was randomly collected from each of the three independent plants, and the three flowers were pooled for genomic DNA extraction. For RNA analysis, three flowers were randomly collected from each independent plant and pooled within each plant for RNA extraction. Thus, each RNA biological replicate represented pooled material from one independent plant, and the reported expression values were calculated from three independent biological replicates.

### 4.2. Paraffin Sectioning and Hematoxylin Staining

Paraffin sections were prepared according to a well-established plant histological protocol with minor modifications [36]. Briefly, fresh floral tissues were harvested and immediately fixed in FAA solution (5% formaldehyde, 5% acetic acid, and 60% ethanol) for 24 h. The fixed samples were sequentially dehydrated in a graded ethanol series (60%, 70%, 80%, 90%, 95%, and 100%) and cleared with a series of ethanol–toluene transparent agent mixtures at volume ratios of 2:1, 1:1, 1:2, and 0:3. Each dehydration and clearing step was subjected to 20 min of vacuum infiltration to ensure sufficient solution penetration. Subsequently, the processed tissues were embedded in paraffin with a melting point of 52 °C and serially sectioned at a thickness of 10 μm using a Leica RM2255 automatic microtome (Nußloch, Germany).

Section staining was performed with hematoxylin according to a published protocol with slight modifications [37]. Briefly, the sections were sequentially rehydrated through a reverse-graded ethanol series, with each treatment lasting 2 min. Subsequently, the sections were mordanted in 4% ammonium ferric sulfate solution for 4 min and stained with 0.1% hematoxylin solution for 10 min. After staining, the sections were differentiated in 1% acetic acid for 30 s and destained in 2% ammonium ferric sulfate solution for 2 min, with gentle rinsing under slow running water for 1 min after each step. Finally, the stained sections were mounted with 50% neutral resin and observed and photographed using a Leica E200 light microscope (Nußloch, Germany).

### 4.3. Extraction of Plant Nucleic Acids

For transcript expression analysis, samples from female, bisexual, and male flowers were collected at anatomically comparable reproductive developmental stages. Although the external size parameters differed among floral types due to differences in organ morphology, the developmental stages were matched according to reproductive cell development, including embryo sac progression in female/bisexual flowers and microspore development in male flowers.

Total RNA Extraction: Remove 1 g of plant tissue from a −80 °C freezer; the plant tissue consists of a mixture of three flowers from three separate plants. The plant tissue is obtained by randomly selecting three flowers from each separate plant and mixing them; grind the mixture into a powder in liquid nitrogen, add 1 mL of Tiangen Triz01A solution (Beijing, China), and lyse for 5 min at 25 °C. The homogenate was centrifuged at 12,000 rpm for 1 min at 4 °C, and 900 μL of the resulting supernatant was transferred to a new centrifuge tube. After the addition of 200 μL chloroform and vigorous mixing, the mixture was centrifuged at 12,000 rpm for 10 min for phase separation. A total of 400 μL of the upper aqueous phase was collected, mixed thoroughly with 200 μL absolute ethanol, and loaded onto an RNA purification column. Following centrifugation at 12,000 rpm for 1 min, the flow-through was discarded. The column was washed with 700 μL of 75% ethanol and centrifuged again at 12,000 rpm for 1 min. Finally, 50 μL of RNase-free water was added to the column for RNA elution via centrifugation at 12,000 rpm for 1 min. The integrity and purity of extracted total RNA were verified by agarose gel electrophoresis and an Agilent UV spectrophotometer (Thermo Fisher Scientific, Santa Clara, CA, USA). High-quality RNA samples were used for subsequent cDNA synthesis and qRT-PCR analysis.

For genomic DNA extraction, total genomic DNA was isolated from floral tissues using a modified CTAB method described by Murray et al., followed by purification with a silica membrane column [38]. Briefly, remove 1 g of plant tissue from a −80 °C freezer; the plant tissue consists of a mixture of three flowers from three separate plants. Grind the tissue into a powder in liquid nitrogen, then add 1 mL of the CTAB extraction solution. The mixture was incubated in a 65 °C water bath for 40 min. After centrifugation at 12,000 rpm for 1 min, 900 μL of the supernatant was transferred to a new tube, mixed thoroughly with 200 μL chloroform, and centrifuged at 12,000 rpm for 10 min. Subsequently, 400 μL of the upper supernatant was collected and blended with 200 μL absolute ethanol and 200 μL saturated sodium chloride solution. The mixed solution was loaded onto a TIANGEN DNA purification column and centrifuged at 12,000 rpm for 1 min, after which the flow-through was discarded. The column was rinsed with 700 μL of 75% ethanol and re-centrifuged under the same conditions. Finally, 50 μL of RNase-free water was applied for DNA elution via 1 min centrifugation at 12,000 rpm. The quality and purity of the extracted genomic DNA were evaluated using agarose gel electrophoresis and an Agilent UV spectrophotometer (Santa Clara, CA, USA).

### 4.4. RT-qPCR Analysis

Floral samples from female flowers treated with 260 mg/L AgNO_3_ and corresponding controls were collected for gene expression analysis. First-strand cDNA was synthesized using 5 μg of qualified total RNA as the template, with the RevertAid First-Strand cDNA Synthesis Kit (Thermo Fisher Scientific, Waltham, MA, USA; Cat. No. K1622) following the manufacturer’s standard protocols. Oligo(dT)_18_ was used as the reverse-transcription primer, and the reaction was performed at 42 °C for 60 min to complete cDNA synthesis.

Subsequent real-time quantitative PCR (RT-qPCR) was conducted on a LightCycler 96 Real-Time PCR System (F. Hoffmann-La Roche AG, Basel, Switzerland) using the SYBR Green fluorescent quantitative method. A total reaction volume of 25 μL was prepared according to the manufacturer’s guidelines of the Maxima SYBR Green qPCR Master Mix (2×) (Thermo Fisher Scientific, Waltham, MA, USA). The PCR amplification procedure consisted of an initial denaturation at 95 °C for 5 min, followed by 30 cycles of 95 °C for 10 s (denaturation) and 60 °C for 45 s (annealing and extension). Following amplification, melting curve analysis was performed using the instrument’s default program to validate the specificity of PCR products. All primer sequences and detailed reaction components are listed in Table S5. The relative expression levels of target genes were quantified via the 2^−ΔΔCt^ method, with the *Actin* gene selected as the internal reference for expression normalization.

### 4.5. Methylation-Sensitive Amplification Polymorphism (MSAP) Analysis

Genomic DNA was isolated from whole female flowers and 260 mg/L AgNO_3_-induced bisexual flowers collected at the flowering stage. Because the MSAP analysis was performed using whole-flower tissues, the resulting methylation profiles represent the composite methylation patterns of the sampled floral tissues. For MSAP analysis, the DNA samples were subjected to parallel double digestion with two restriction enzyme combinations: EcoRI/HpaII and EcoRI/MspI. Pre-amplification was performed using adapter-specific primers (Table S6), followed by selective amplification with 50 distinct primer pairs (Table S7). All PCR amplification reactions and thermal cycling profiles were performed in accordance with the manufacturer’s protocols for Kangwei Omnipotent PCR Mix (Jiangsu CoWin Biotech Co., Ltd., Taizhou, China). For electrophoretic detection, 6 μL of selective amplification products was separated via 6% non-denaturing polyacrylamide gel electrophoresis (PAGE) at a constant power of 70 W for 2 h. The gel was subsequently stained with AgNO_3_ solution (0.75 g/L) and developed using a sodium hydroxide developer (30 g/L NaOH supplemented with 2 mL/L formaldehyde). The resulting DNA banding profiles were visualized and recorded for subsequent methylation pattern analysis. Among the tested concentrations, 260 mg/L AgNO_3_ exhibited the strongest induction effect on bisexual flower formation and was therefore selected for subsequent molecular analyses, including qRT-PCR and MSAP assays. One pooled DNA sample was generated from three flowers of three independent plants.

### 4.6. Statistical Analysis

All morphological measurements, RT-qPCR data, and MSAP profiling results in this study were statistically analyzed using SPSS 20.0 software. One-way analysis of variance (ANOVA) followed by Tukey’s HSD multiple comparison test was performed to evaluate significant differences among experimental groups. A *p*-value less than 0.05 was defined as the threshold for statistical significance. All quantitative graphs were generated using GraphPad Prism software (version 10.4.1).

## Figures and Tables

**Table 1:** Statistical summary of MSAP methylation band profiles in female and AgNO_3_-induced bisexual flowers of *Siraitia grosvenorii*.

Banding Patterns(H, M)	Female Flower	Bisexual Flower	DNA Methylation Site
A(−, +)	348 (16.03%)	380 (17.06%)	Internal cytosine methylation
B(+, −)	231 (10.64%)	182 (8.17%)	Hemimethylation or internal cytosine methylation
C(+, +)	1563 (71.99%)	1546 (69.42%)	Absence of methylation
D(−, −)	29 (1.34%)	119 (5.34%)	Complete double-stranded methylation

## Data Availability

The original contributions presented in this study are included in the article/Supplementary Materials. Further inquiries can be directed to the corresponding authors.

## Supplementary Materials

The following supporting information can be downloaded at: https://www.mdpi.com/article/10.3390/plants15182796/s1, Table S1. Megasporogenesis. Table S2. Microspore and pollen. Table S3. Methylation loci. Table S4. Methylation alteration. Table S5. Pre-amplification primer sequences, reaction system and PCR program. Table S6. MSAP adapter sequences. Table S7. The primers of selective amplification. Figure S1. Electrophoretic profiles of five selective primer combinations (E1/HM7, E1/HM8, E4/HM5, E4/HM7, and E4/HM8) used in MSAP analysis.

## Abbreviations

*S. grosvenorii*	*Siraitia grosvenorii*
PMC	Pollen mother cell
MSAP	Methylation-sensitive amplification polymorphism
AgNO_3_	Silver nitrate
ACS	1-aminocyclopropane-1-carboxylate synthase
SAM	S-adenosylmethionine
ACO	1-aminocyclopropane-1-carboxylate oxidase
PCD	Programmed cell death
RT-qPCR	Real-time quantitative PCR
PAGE	Polyacrylamide gel electrophoresis
